# Consanguinity-Associated Genetic Disorders in Saudi Arabia: A Hypothesis on the Future Role of CRISPR-Based Gene Editing

**DOI:** 10.7759/cureus.113445

**Published:** 2026-07-27

**Authors:** Dana Aldughim

**Affiliations:** 1 Medicine, University of Glasgow, Glasgow, GBR

**Keywords:** consanguineous marriage, crispr gene editing, genetic disorders, public awareness, saudi arabia

## Abstract

Background

Consanguineous marriage remains highly prevalent in Saudi Arabia and is strongly associated with increased rates of inherited genetic disorders, including sickle cell disease and β-thalassemia. The persistence of such marriages, driven by cultural and social factors, limits the effectiveness of existing preventive strategies such as premarital screening and genetic counselling.

Methodology

A cross-sectional, survey-based study was conducted between November and December 2025 among 139 participants across Saudi Arabia. The questionnaire was distributed via WhatsApp messaging groups and administered using Google Forms. The 17-item survey covered CRISPR awareness, perceptions of consanguinity and genetic disease risk, and attitudes toward CRISPR as a therapeutic intervention. Data were analysed using descriptive statistics.

Results

Of 139 participants, 60.4% had previously heard of CRISPR; however, 55.4% demonstrated limited or no meaningful knowledge of the technology. Despite this, 96.4% expressed willingness to accept CRISPR-based treatment for inherited genetic disorders. Scientific safety, religious considerations, and cost were the most frequently cited factors influencing acceptance.

Conclusions

Public awareness of CRISPR in Saudi Arabia remains critically low despite high receptiveness to its therapeutic applications. Integrating targeted public education with CRISPR-based somatic cell interventions could represent an effective long-term strategy for reducing the burden of inherited genetic disorders associated with consanguineous marriage in Saudi Arabia.

## Introduction

Genetic disorders represent an increasing global public health challenge, with consequences that extend beyond medical complications to include significant psychological, social, and economic burdens [1]. Globally, rare genetic disorders place a substantial economic burden on healthcare systems worldwide, extending well beyond direct medical costs [1]. Conventional cost-of-illness analyses often underestimate this burden by excluding indirect costs such as emotional distress, loss of productivity, and long-term caregiving responsibilities [1]. Furthermore, severe and progressive inherited conditions underscore the importance of accurate prevalence data, as their significant physical and emotional impact often motivates families with a known genetic background to pursue genetic counselling to reduce the risk of recurrence [2]. In Saudi Arabia, inherited genetic disorders are observed at relatively higher rates than in many other regions, largely due to the widespread practice of consanguineous marriage, which significantly increases the risk of autosomal recessive conditions [3,4]. Although current preventive strategies such as premarital screening and genetic counselling have contributed to increased awareness, their effectiveness in reducing long-term disease prevalence remains limited [3].

Among emerging solutions, CRISPR-Cas systems offer a highly efficient and cost-effective approach to correcting disease-causing mutations, primarily through somatic cell gene editing, which modifies non-heritable cells in the patient only rather than germline editing, which would affect all future generations and raises significant ethical and regulatory concerns [5]. Recent developments have further improved the precision and safety of these systems [5]. Recent advances in translational genomics and personalised medicine further highlight the importance of integrating emerging genetic technologies into public health strategies, reinforcing the need for greater public awareness of tools such as CRISPR [6]. However, it remains unclear whether the limited effectiveness of current strategies reflects cultural persistence alone or also gaps in public awareness of technologies such as CRISPR, forming the basis of the present hypothesis.

Hypothesis

This study proposes that the limited effectiveness of current preventive strategies in Saudi Arabia is not solely due to the persistence of consanguineous marriage but also due to insufficient public awareness of advanced genetic technologies such as CRISPR. It is hypothesised that integrating gene-editing interventions with targeted public education could significantly improve disease prevention outcomes and reduce the long-term burden of inherited genetic disorders.

## Materials and methods

A cross-sectional survey-based study was conducted between November and December 2025 to explore public awareness and perceptions of CRISPR gene-editing technology in Saudi Arabia, in the context of consanguineous marriage and inherited genetic disorders. The study was conducted online and targeted participants residing across multiple regions of Saudi Arabia, including urban and suburban areas. No specific geographic region was excluded, and participants were recruited from diverse locations across the Kingdom.

The survey instrument was developed by the author in late November 2025 and distributed online throughout December 2025 over a period of approximately one month. The questionnaire was shared primarily via WhatsApp messaging groups, targeting a broad and diverse audience across Saudi Arabia, with particular outreach to university students from various academic disciplines, including science, health sciences, arts, and administrative fields. Participants were informed at the beginning of the survey that participation was entirely voluntary, that no personal identifying information would be collected, and that responses would remain fully anonymous.

The survey questions were developed by the author based on a review of the existing literature on CRISPR awareness and consanguinity. The questionnaire was not formally validated by external experts, as this study was designed as a preliminary, exploratory investigation. Future studies may benefit from expert validation of survey instruments to enhance reliability.

A total of 139 participants completed the survey, representing a range of demographic backgrounds with variation in age, gender, educational level, and field of study. Inclusion criteria required participants to be residents of Saudi Arabia at the time of the survey. Incomplete or partially submitted responses were excluded from the analysis to ensure data integrity.

The questionnaire consisted of 17 items divided into the following four sections: demographic information, CRISPR awareness, perceptions of consanguinity and genetic disease risk, and attitudes toward CRISPR as a therapeutic intervention. Questions were presented in Arabic to ensure accessibility across participant groups. The survey was administered using Google Forms, which served as the primary data collection and management tool throughout the study.

Data were analysed using descriptive statistics. Responses were summarised as frequencies (n) and percentages (%) to highlight overall patterns in awareness and attitudes among participants. No inferential statistical tests were applied, as the study aimed to provide a descriptive overview of public knowledge and attitudes rather than test causal hypotheses. Ethical compliance was maintained throughout; the study involved no clinical intervention, no collection of personal data, and participation was fully voluntary and anonymous.

## Results

A cross-sectional survey was completed by 139 participants from diverse demographic backgrounds across Saudi Arabia, including individuals varying in age, gender, and educational level. The findings revealed notable patterns in public awareness of CRISPR gene-editing technology and attitudes toward its application in addressing inherited genetic disorders.

Demographic characteristics of participants

The demographic characteristics of the 139 survey participants are summarised in Table 1. The majority of the respondents were aged 18-24 years (69.8%), female (86.9%), enrolled as bachelor’s students (74.1%), and from scientific disciplines (65.5%).

**Table 1:** Demographic characteristics of survey participants (n = 139).

Characteristic	Category	n	%
Age	Under 18	3	2.2%
	18–24	97	69.8%
	25–30	22	15.8%
	Over 30	17	12.2%
Gender	Female	119	86.9%
	Male	18	13.1%
Educational level	Bachelor’s student	103	74.1%
	Postgraduate student	7	5.0%
	Other	29	20.9%
Field of study	Science	91	65.5%
	Health	12	8.6%
	Arts/Administration	16	11.5%
	Other	20	14.4%

Awareness of CRISPR technology

Participants were asked whether they had previously heard of CRISPR gene-editing technology. As presented in Table 2, the majority of respondents (60.4%, n = 84) reported having heard of CRISPR, while 38.1% (n = 53) had no prior awareness of the technology.

**Table 2:** Frequency of participants who had heard of CRISPR gene-editing technology prior to the survey (n = 139).

Response	n	%
Heard of CRISPR	84	60.4%
Not heard of CRISPR	53	38.1%
Total	139	100%

Despite the majority having heard of CRISPR, self-assessed knowledge levels were predominantly low, as illustrated in Table 3. Specifically, 44.6% of respondents indicated they had no knowledge of CRISPR, and a further 10.8% stated they only recognised the name without understanding its function. Consequently, 55.4% of all participants demonstrated limited or no meaningful awareness of the technology. Only 14.4% reported good knowledge, and a mere 2.2% described themselves as having deep knowledge of CRISPR. Among those who had heard of CRISPR, the most commonly cited sources were social media, university lectures, and online articles, suggesting that formal academic exposure plays a role but remains insufficient in reaching the broader population.

**Table 3:** Self-assessed CRISPR knowledge levels among survey participants (n = 139). The majority reported no knowledge (44.6%) or recognised the name only (10.8%), yielding a combined limited awareness rate of 55.4%.

Knowledge level	n	%	Cumulative %
No knowledge whatsoever	62	44.6%	44.6%
Recognise name only	15	10.8%	55.4%
Basic knowledge	37	26.6%	82.0%
Good knowledge	20	14.4%	96.4%
Deep knowledge	3	2.2%	98.6%
Total	139	100%	

Perceptions of consanguinity and genetic disease risk

Participants were asked whether they believed consanguineous marriage increases the likelihood of inherited genetic disorders. As shown in Table 4, the majority of respondents agreed that consanguinity poses a genetic risk, while a notable proportion remained uncertain.

**Table 4:** Participant responses to whether consanguineous marriage increases the likelihood of inherited genetic disorders (n = 139).

Response	n	%
Yes — consanguinity increases risk	109	78.4%
Unsure	25	18.0%
No	5	3.6%
Total	139	100%

With respect to consanguineous marriage and its association with genetic disorders, 78.4% of participants agreed that consanguinity increases the likelihood of inherited diseases. However, 18.0% expressed uncertainty, and 3.6% did not believe such a relationship exists. These findings indicate that while general awareness of the genetic risks associated with consanguinity is relatively high, a substantial proportion of the population remains uncertain, which may partially explain why preventive strategies have had limited behavioural impact despite increased screening efforts.

Attitudes toward CRISPR as a therapeutic intervention

Participants were asked whether they would accept CRISPR-based treatment for an inherited genetic disorder in a family member. As presented in Table 5, the overwhelming majority expressed willingness to accept such treatment, reflecting markedly positive attitudes toward the clinical application of CRISPR despite overall low knowledge levels.

**Table 5:** Participant willingness to accept CRISPR-based treatment for an inherited genetic disorder in a family member (n = 139). Combined acceptance rate (yes + maybe) was 96.4%.

Response	n	%	Cumulative acceptance %
Yes, definitely	89	64.3%	64.3%
Maybe	45	32.4%	96.4%
No	5	3.6%	—
Total	139	100%	

Despite the overall low levels of CRISPR knowledge, participant attitudes toward its medical application were markedly positive. Specifically, 64.3% responded “yes, definitely” and 32.4% responded “maybe,” yielding a combined acceptance rate of 96.4%. Only 3.6% of participants indicated they would not consider such treatment.

Factors influencing acceptance and perceived applications

Participants were asked to identify the factors that most influenced their acceptance of CRISPR technology. As presented in Table 6, scientific safety was the most frequently cited concern, followed by religious considerations, cost, and lack of information.

**Table 6:** Factors reported by participants as influencing their acceptance of CRISPR gene-editing technology (n = 139).

Factor	n	% of respondents
Scientific safety	95	68.3%
Religious considerations	82	59.0%
Cost	78	56.1%
Lack of information	72	51.8%
Ethical concerns	65	46.8%
Social acceptance	35	25.2%

Participants were also asked to identify which sectors in Saudi Arabia could most benefit from CRISPR technology. As illustrated in Table 7, the healthcare sector was overwhelmingly identified as the primary field, followed by scientific research and agriculture.

**Table 7:** Sectors identified by participants as most likely to benefit from CRISPR technology in Saudi Arabia (n = 139). Multiple responses were permitted.

Sector	n	% of respondents
Healthcare	118	84.9%
Scientific research	89	64.0%
Agriculture	67	48.2%
Food security	54	38.8%
Cosmetic medicine	42	30.2%
Other	38	27.3%

Among the factors reported as influencing participants’ acceptance of CRISPR, scientific safety was the most frequently cited concern, followed by religious considerations, ethical issues, cost, and limited available information. These results indicate that public hesitancy is not necessarily grounded in moral rejection of the technology but rather in insufficient knowledge and concerns about its reliability and accessibility. Regarding potential applications, the healthcare sector was overwhelmingly identified as the primary field that could benefit from CRISPR in Saudi Arabia, with scientific research and food security also frequently mentioned.

Overall, these results support the study’s central hypothesis. The data demonstrate that awareness of CRISPR remains low across the sampled population, even among those who have heard the term, suggesting that limited public education is a key barrier to the broader adoption of gene-editing approaches. At the same time, the high levels of acceptance indicate that, once informed, the Saudi public is open to integrating CRISPR into the national healthcare strategy.

## Discussion

Global burden of genetic disorders and emerging genetic solutions

Neurological and genetic disorders represent a growing global health burden, contributing significantly to mortality and long-term disability worldwide [7]. Many of these conditions are chronic and progressive, meaning that they worsen over time and often require continuous medical care and long-term management. Rare diseases, in particular, place a considerable socioeconomic burden on patients, families, healthcare systems, and society as a whole, extending beyond direct medical expenses to include wider social and economic consequences [1]. Moreover, neurological disorders affect billions of people globally and represent a major contributor to both disability and mortality worldwide [7]. This issue is more pronounced in countries such as Saudi Arabia, where consanguineous marriage contributes to higher rates of inherited genetic disorders. A recent population-based cohort study from the Middle East further demonstrated that consanguinity is significantly associated with adverse fetal outcomes, highlighting the urgent need for integrated public health interventions that go beyond conventional screening approaches [8]. These challenges have driven growing interest in emerging genetic solutions, including gene therapy and CRISPR-based interventions, which offer promising avenues for addressing the underlying causes of inherited disorders rather than managing their symptoms alone [5].

Consanguineous marriage as a key driver of genetic disorders in Saudi Arabia

Consanguineous marriage in Saudi Arabia is strongly influenced by cultural and social factors. Saudi Arabia exhibits one of the highest consanguinity rates in the region, exceeding many neighbouring countries. In many Middle Eastern societies, marriage between relatives, particularly first cousins, is widely accepted due to cultural, social, and economic reasons [9]. Studies in Saudi Arabia have consistently reported a high prevalence of first-cousin marriages, with rates remaining stable at approximately 57.7% over several decades, indicating little change across generations [9]. The preference for such marriages is largely driven by sociocultural factors, including the preservation of family structure and social relationships [4].

From a genetic perspective, consanguineous marriages increase the probability of autosomal recessive disorders, as related individuals are more likely to share identical genetic mutations [4]. When both parents carry the same recessive mutation, there is a higher likelihood that their child will inherit two copies of the defective gene, leading to the expression of a genetic disorder. This relationship is further supported by evidence indicating a high prevalence of inherited disorders in Saudi Arabia, with consanguinity identified as a key factor influencing patterns of inheritance and disease distribution [3].

Genetic conditions such as sickle cell disease are highly prevalent in Saudi Arabia and represent a significant public health concern [10]. As an autosomal recessive disorder, sickle cell disease is associated with a wide range of complications and requires long-term medical care [10]. While Saudi Arabia has introduced preventive measures such as mandatory premarital screening programmes, others, including sickle cell disease, remain highly prevalent, particularly in rural areas where consanguineous marriage is more common [9].

Effectiveness and limitations of current preventive strategies in Saudi Arabia

The Saudi Premarital Screening and Genetic Counselling Programme, now known as the Healthy Marriage Programme, was implemented in 2004 and initially targeted sickle cell disease and β-thalassemia, later expanding to include HIV and hepatitis B and C [3,9]. The Saudi government has introduced national screening programmes; however, these programmes currently cover only a limited number of conditions [3]. A five-year study assessing the impact of premarital screening found that the rate of β-thalassemia decreased following the implementation of screening programmes [9], demonstrating that such strategies can produce measurable benefits when effectively implemented.

However, cultural and social factors play a significant role, as many individuals continue to engage in consanguineous marriage despite being aware of the associated genetic risks [3]. This indicates that awareness alone is not sufficient to influence behaviour. In addition, genetic counselling services are often limited by low levels of public awareness and restricted access, meaning that not all individuals fully understand or utilise these services [2]. Overall, while current strategies focus on risk identification and awareness, they do not directly address the underlying genetic causes of disease, highlighting the need for more advanced approaches such as CRISPR-based gene editing. Studies from neighbouring countries in the region have similarly highlighted limited awareness and mixed attitudes toward premarital screening, suggesting that cultural and social barriers to genetic disease prevention extend beyond Saudi Arabia [11].

In the view of the present author, CRISPR-based interventions offer a practical advantage over existing preventive strategies in the Saudi context precisely because they address the underlying genetic cause of disease rather than relying on behavioural change. Given that approximately 88% of at-risk couples in Saudi Arabia proceed with marriage even after receiving positive screening results [9], strategies dependent on voluntary behaviour modification have demonstrated clear limitations. CRISPR, by contrast, offers the possibility of treating or correcting inherited mutations in affected individuals regardless of marital choices, making it a complementary and potentially more impactful long-term solution for reducing the burden of inherited genetic disorders in Saudi Arabia.

CRISPR gene editing as an innovative therapeutic approach

CRISPR gene editing has emerged as a promising therapeutic approach for inherited genetic disorders because it directly targets and modifies disease-causing mutations [5]. This technology relies on the Cas9 enzyme and a guide RNA to recognise specific DNA sequences and create precise cuts, allowing cellular repair mechanisms to correct or replace faulty genes [5,12]. In the context of this study, CRISPR-based gene editing is discussed primarily as a somatic cell therapy, targeting non-reproductive cells in an individual patient, rather than germline editing, which would modify heritable DNA and affect all future generations. While germline editing remains ethically contentious and is subject to strict international regulatory oversight, somatic CRISPR therapies are currently the focus of clinical trials and have received regulatory approval for conditions such as sickle cell disease [5,12]. One of the main advantages of CRISPR is its high precision, efficiency, and relatively low cost compared to earlier gene-editing technologies [5,13]. CRISPR has already been explored in gene therapy for several inherited conditions, including sickle cell disease, β-thalassemia, cystic fibrosis, and muscular dystrophy [12].

The relevance of CRISPR is particularly significant in Saudi Arabia, where inherited genetic disorders are highly prevalent due to the high rate of consanguineous marriage [3,9]. Introducing CRISPR-based therapies could provide a more effective long-term strategy for reducing the burden of inherited diseases associated with consanguinity. However, CRISPR still faces important limitations, including the risk of off-target effects, ethical concerns, and challenges related to the safe delivery of CRISPR components into cells [12,13]. Broader reviews of the literature support these findings. A systematic review by Ramos et al. [14] examining 53 survey studies across four continents found that public acceptance of gene-editing is consistently higher for therapeutic applications than for enhancement purposes. Similarly, Ronanki and Sivala [15] reported that public awareness of CRISPR remains limited across diverse populations, with acceptance largely dependent on the perceived safety and medical necessity of the intervention. Together, these studies suggest that the pattern observed in Saudi Arabia of low awareness yet high therapeutic acceptance is not unique, but reflects a broader global trend that underscores the urgent need for targeted public education campaigns.

Limitations

Despite the contributions of this study, several limitations should be acknowledged. First, the sample size of 139 participants is relatively small and may not be fully representative of the broader Saudi population. Second, the survey was distributed exclusively through online social media platforms, which may have introduced a selection bias by excluding individuals with limited internet access, particularly those from rural areas where consanguineous marriage rates tend to be higher. Third, knowledge of CRISPR technology was assessed through self-report rather than objective testing, meaning that participants’ ratings of their own knowledge may not accurately reflect their actual understanding of the technology. Fourth, as participants self-selected to complete the survey, the sample may over-represent individuals with a prior interest in science or genetics, potentially inflating awareness levels. Finally, the cross-sectional design of this study does not allow for the establishment of causal relationships between public awareness and health outcomes. Future studies with larger, more representative samples and longitudinal designs would be needed to validate and expand upon these findings.

## Conclusions

Inherited genetic disorders remain a significant public health concern, particularly in Saudi Arabia, where consanguineous marriage continues to play a major role in increasing their prevalence. It is important to emphasise that avoiding consanguineous marriage, together with premarital genetic screening and genetic counselling, remains the primary and most effective population-level strategy for reducing the burden of inherited genetic disorders. These approaches address risk at the source and should continue to be prioritised and strengthened within Saudi Arabia’s public health framework. Although their overall impact remains limited by cultural and behavioural factors, as they do not address the underlying genetic causes of disease in affected individuals, they represent the foundation of any prevention strategy. This study has critically examined how consanguineous marriage contributes to the persistence of inherited genetic disorders in Saudi Arabia and has evaluated the potential of CRISPR gene-editing technology as a complementary innovative approach. CRISPR offers a more advanced and targeted solution by directly modifying disease-causing mutations in affected patients through somatic cell therapy; however, challenges such as ethical concerns, off-target effects, and delivery limitations still restrict its widespread clinical application. Therefore, CRISPR should be regarded not as a replacement for established preventive strategies, but as a complementary therapeutic tool whose future effectiveness will depend on continued scientific progress, targeted public education, and careful ethical consideration.

## Appendices

Appendix A: Survey questionnaire

The following questionnaire was developed by the author and distributed online in Arabic across Saudi Arabia in December 2025. Translation provided below.

**Table 8:** Demographic information.

Q#	Question	Response options
Q1	Age	Under 18/18–24/25–30/Over 30
Q2	Gender	Male/Female
Q3	Educational level	Bachelor’s student/Postgraduate student/Other
Q4	Field of study	Science (Biology, Physics, Mathematics, CS)/Health (Medicine, Nursing, Pharmacy)/Arts/Administration/Other

**Table 9:** CRISPR awareness questions.

Q#	Question	Response options
Q5	Have you previously heard of CRISPR technology?	Yes/No
Q6	What is your source of knowledge about CRISPR?	Social media/University lectures/Internet articles/Friends & family/Never heard of it
Q7	How would you rate your knowledge of CRISPR?	No knowledge/Name only/Basic/Good/Deep knowledge

**Table 10:** Consanguinity and genetic disease questions.

Q#	Question	Response options
Q8	Do you believe consanguineous marriage increases the likelihood of inherited genetic disorders?	Yes/No/Unsure
Q9	Do you have a family member with a genetic disorder that may be linked to consanguineous marriage?	Yes/No/Prefer not to answer

**Table 11:** CRISPR attitudes and applications questions.

Q#	Question	Response options
Q10	I believe CRISPR can help reduce genetic disorders linked to consanguineous marriage.	Yes/No
Q11	I have ethical concerns about genetic manipulation of human DNA.	Yes/No
Q12	I agree to using CRISPR only to treat serious genetic disorders.	Yes/No
Q13	I reject CRISPR for cosmetic purposes (e.g. height, eye colour).	Yes/No
Q14	I believe Saudi Arabia should invest in CRISPR research.	Yes/No
Q15	If a CRISPR-based treatment were available for an inherited disorder in your family, would you accept it?	Yes, definitely/Maybe/No
Q16	What factors influence your acceptance of CRISPR?	Scientific safety/Religious concerns/Ethical concerns/Social acceptance/Cost/Lack of information (multiple choice)
Q17	Which sectors could most benefit from CRISPR in Saudi Arabia?	Healthcare/Agriculture/Scientific research/Cosmetic medicine/Food security/Other (multiple choice)
